# Sample size estimation practices in research protocols submitted to Danish scientific ethics committees

**DOI:** 10.1016/j.conctc.2018.08.003

**Published:** 2018-08-15

**Authors:** Marius Mølsted Flege, Simon Francis Thomsen

**Affiliations:** a Department of Dermatology, Bispebjerg Hospital, Copenhagen, Denmark; b Department of Biomedical Sciences, University of Copenhagen, Copenhagen, Denmark

## Abstract

**Background:**

Sample size in research projects is estimated before initiation of the study to minimise type 1 and type 2 error, while keeping the study's financial cost and subject enrolment to a minimum. This study investigates project-specific factors potentially associated with correct estimation of sample size in study protocols.

**Methods:**

Examination of 189 non-commercially sponsored study protocols (84 randomised controlled trials (RCTs) and 105 non-RCT studies) submitted to the Scientific Ethics Committees of The Capitol Region of Denmark from 2013 to 2015.

**Results:**

119 (63%) study protocols contained a sample size calculation, with a significantly higher rate of sample size calculations in RCT vs non-RCT study protocols (76% vs. 52%, p < 0.001). Significantly more intervention studies than non-intervention studies (69% vs 52%, p = 0.020), studies including blood samples compared to those without (69% vs. 55%, p = 0.045), studies funded by a foundation donation compared to those with no funding (68% vs. 49%, p = 0.040) performed sample size calculations. Further, increasing number of sick patients enrolled (p = 0.048) and newer studies (p = 0.032) were more likely to include a sample size calculation in the protocol.

**Conclusions:**

Estimation of sample size is more often reported in RCT than non-RCT study protocols. Also, intervention studies, studies funded by a foundation donation, studies including blood samples, studies with a greater amount of sick participants and chronologically newer study protocols more often reported a sample size calculation.

## 1 Introduction

Estimation of projected sample size of a medical research study is based on the hypothesis proposed by the investigators. For various reasons investigators sometimes fail to determine sample size before initiation of the study and consequently base their projected number of participants on experience, logistics or something even more unscientific [1]. A sample size too small increases the risk of type 2 error, whereas a study with too many participants potentially exposes research subjects to unnecessary risk and uses up excess resources [2].

Previous studies have explored underpowered designs and how the concept of statistical power can be misused and is often misunderstood [[2], [3], [4]]. Recent studies have investigated sample size estimation empirically [1,5,6]. Notably, in regards to randomised controlled trials (RCTs) conducted in the UK one study from 2013 reported that only 42% of the protocols supplied all the information needed to repeat the sample size calculation. Non-commercially sponsored studies were less likely to report all the information and researchers tended to overestimate their sample size [1]. A French study from 2009 found that among selected MEDLINE RCT-articles, 43% did not present enough information to repeat the sample size calculation, while 5% did not report conducting a sample size calculation at all [5].An American study from 2015 showed that only 56% of clinical trials of analgesics reported a sample size calculation, but just 38% included all the parameters needed to recalculate the sample size. The authors also found that RCTs reported a sample size calculation more often than other experimental study types [6].

To extend previous knowledge, the aim of this study was to investigate sample size estimation practices and use of statistical sample size calculation in non-commercially sponsored original research protocols submitted to the Scientific Ethics Committees of The Capitol Region of Denmark. Specifically, we identified project-specific indicators possibly associated with correct or incorrect statistical sample size calculation and sample size estimation.

## 2 Methods

### 2.1 Identification of research protocols

In Denmark, all original research protocols are submitted to one of five central Scientific Ethics Committees corresponding to the five geographical regions in the country. One of these regions is the Capitol Region (greater Copenhagen), which receive approximately 700 original research protocols annually (an annual mean of 721 through 2013–2017). This number is roughly equal to the total number of research protocols evaluated by the other four Regional Scientific Ethics Committees combined.

We identified non-commercially sponsored study protocols submitted from January 2013 onwards and included, consecutively, all protocols until July 2015 when 89 RCT protocols were ascertained. Only non-commercial study protocols were included to ensure that sample size calculation was not performed or included in the study design by a commercial sponsor before the academic investigators were involved in the study design. Commercially sponsored studies usually include a power calculation as per company tradition, and therefore may not reflect the individual academic investigators' practices. During this period 100 random non-RCT protocols were included for comparison. Inclusion of the earliest versions of the protocols was prioritized to more reliably represent the original research intention of the investigators. As per Danish law research protocols must include some considerations about participant number but not necessarily a mathematical sample size calculation. This sampling strategy was based on the assumption that, in order to detect at least a 20% difference in the prevalence of reported sample size estimation in two groups (our primary study endpoint); RCT protocols = 75% and non-RCT protocols = 55%, 86 protocols were needed in each category with a power of 80% (β = 0.2) at the 95% significance level (α = 0.05) in the two groups.

### 2.2 Data extraction

For each study protocol data were extracted from the protocol report formula, the statistical subsection of the protocol, and the study participant information, which were the documents covered by the right of access. As per Danish law it is possible for all citizens (option pursued particularly by journalists and other researchers who perform meta-research) to apply for right of access to individual research project documents. The documents concerning this project were delivered to the authors by the administrative personnel of the Scientific Ethics Committees of The Capitol Region of Denmark via secure delivery of PDF (portable document files). The extracted data were organised in a database comprising the following exposure variables: study type (RCT/non-RCT), researcher's academic status (junior/senior), researcher's gender (female/male), institution (state hospital/regional hospital and non-hospital), intervention (treatment/other intervention/none), biobank status (existing biobank/new biobank/none), study participant remuneration (yes/no), multistate study (yes/no), field of medicine (internal medicine/surgery/other), procedures involved (electromagnetic radiation exposure/blood sample/biopsy/none), economic funding (state funded/medicinal company funded/foundation donation funded/no funding), study length (months), participants (number of healthy/sick), study sessions (number), time to new year (weeks), time passed since 2013 (months).

Although study type was stated by the researcher in the protocol report formula, not always did it match the actual study type and subsequently the authors re-classified the study type by evaluating the participant information form and the study description found in the protocol report formula. Of the 89 protocols submitted as RCTs, only 84 were determined to be RCTs when the protocol report formulae and the study participant information were examined by the authors.

### 2.3 Evaluation of sample size estimation

Information on the following components of sample size estimation was extracted from the statistical subsection of the protocols: α (significance level), β (power), expected proportion of drop-outs, effect size (minimal relevant difference, MIREDIF) and standard deviation (or two proportions, if the study compared proportions). Further, an estimation of sample size was performed by the authors based on the reported components and compared to the reported sample size in the protocol.

Whether a sample size calculation was performed by the researcher in the study protocol was our primary outcome. As supporting secondary analysis, we constructed a sample size calculation component composite score (SSCCCS) ranging from 0 to 6 with one point given for enough information conveyed to reproduce correctly the calculation and achieving the same sample size estimate, and one point for each of the following five constants reported; α, β, expected dropout fraction, effect size and standard deviation (or two proportions).

The data analysis was conducted in SPSS version 25, using logistic and linear regression functions.

## 3 Results

**Fig. 1:**
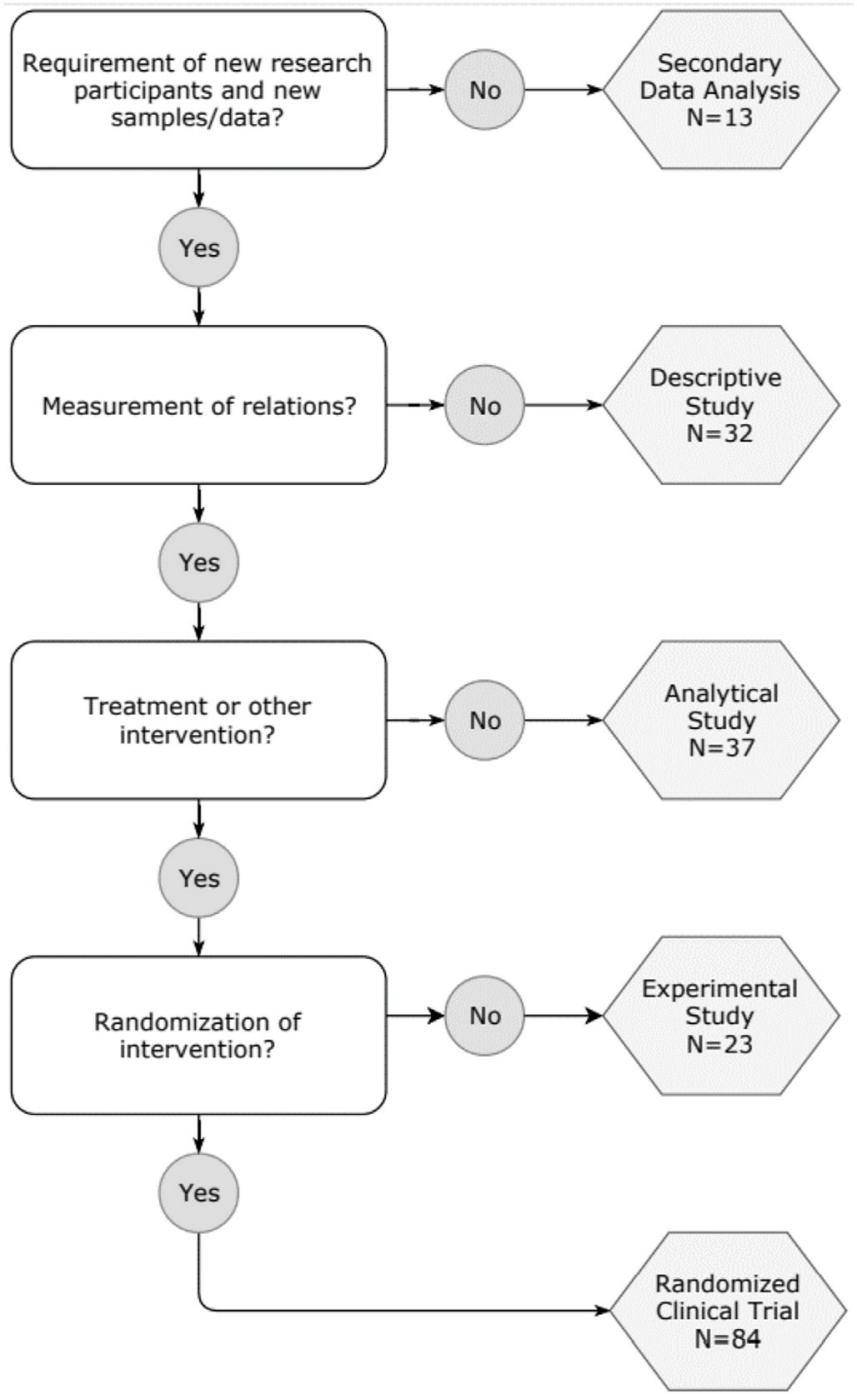
Classification of the included studies. The flowchart describes the authors' algorithm used to divide the studies in the five categories.

In total 189 study protocols were identified and analysed; 84 (44%) were RCTs, 23 (12%) were other type of experimental studies, 37 (20%) were analytical studies, 32 (17%) were descriptive studies, and 13 (7%) were studies including secondary data analysis (e.g. stored tissue samples) (Fig. 1).

The researcher's academic status and gender were senior in 138 (73%) studies and male in 112 (59%) studies. 77 (41%) studies were conducted by the state hospital, while the rest were conducted in regional hospitals and non-hospital institutions. 93 (49%) studies involved treatment, whereas 14 (7%) involved some other interventions. 84 (44%) of the studies were in the field of internal medicine and 24 (13%) were in surgery. 107 (57%) of the studies included blood samples as procedure, 37 (20%) involved electromagnetic radiation exposure (such as X-ray), and 20 (11%) included biopsies. 134 (71%) of the studies were funded by a foundation donation, while, respectively, 8 (4%) and 14 (7%) studies were state funded and medicinal company funded; 33 (18%) had no funding (Table 1).

**Table 1:** Characteristics of the included study protocols.

		RCT N = 84		Non-RCT N = 105		Total N = 189	
Researcher's academic status, N (%)	Junior	24	(28.6)	27	(25.7)	51	(27.0)
	Senior	60	(71.4)	78	(74.3)	138	(73.0)
Researcher's gender, N (%)	Female	35	(41.7)	42	(40.0)	77	(40.7)
	Male	49	(58.3)	63	(60.0)	112	(59.3)
Institution, N (%)	State hospital	31	(36.9)	46	(43.8)	77	(40.7)
	Regional hospital	53	(63.1)	59	(56.2)	112	(59.3)
	Non-hospital	2	(2.4)	1	(1.0)	3	(1.6)
Intervention, N (%)	Treatment	77	(91.7)	14	(13.3)	93	(49.2)
	Other intervention	7	(8.3)	43	(41.0)	14	(7.4)
	None	0	(0.0)	48	(45.7)	82	(43.4)
Biobank, N (%)	Existing biobank	0	(0.0)	15	(14.3)	15	(7.9)
	New biobank	43	(51.2)	49	(46.7)	92	(48.7)
	None	41	(48.8)	44	(39.0)	85	(45.0)
Remuneration, N (%)	Yes	27	(32.1)	29	(27.6)	56	(29.6)
Multistate project, N (%)	Yes	9	(10.7)	10	(9.5)	19	(10.1)
Field of medicine, N (%)	Internal medicine	41	(48.8)	43	(41.0)	84	(44.4)
	Surgery	10	(11.9)	14	(13.3)	24	(12.7)
	Other	33	(33.9)	48	(45.7)	81	(42.9)
Invasive procedures, N (%)	Electromagnetic radiation exposure	17	(20.2)	20	(19.0)	37	(19.6)
	Blood sample	51	(60.7)	56	(53.3)	107	(56.6)
	Biopsy	8	(9.5)	12	(11.4)	20	(10.6)
	None	27	(32.1)	41	(39.0)	68	(36.0)
Economical funding, N (%)	State funded	3	(3.6)	5	(4.8)	8	(4.2)
	Medicinal company funded	7	(8.3)	7	(6.7)	14	(7.4)
	Foundation donation funded	62	(73.8)	72	(68.6)	134	(70.9)
	No funding	12	(14.3)	21	(20.0)	33	(17.5)
Study length, mean (SD)	Months	26	(33)	39	(119)	33	(90)
Participants, median (range)	Total	42	(1492)	70	(5435)	50	(5435)
	Health	0	(450)	10	(3400)	0	(3400)
	Sick	30	(1500)	40	(5441)	40	(5441)
Sessions, mean (SD)	Sessions	4.1	(2.8)	2.6	(3.4)	3.2	(3.3)
Time to new year, mean (SD)	Weeks	19	(9)	14	(9)	16	(10)
Time passed since 2013, mean (SD)	Months	13	(7)	12	(7)	13	(7)
SSCCCS, mean (SD)	Points	4.0	(2.3)	2.6	(2.4)	3.2	(2.5)

Non-RCT: Include experimental, analytical, descriptive and secondary data analysis study types.

Electromagnetic radiation exposure: Include all procedures with x-rays or radioactive isotopes.

Biopsy procedures: Include skin, muscle, liver, fatty tissue, and bone marrow biopsies.

Funding: The main funder of the study.

Time to new year: Measured as the shorter of the two following; number of days that has passed from submit of protocol to new year or number of days from submission of protocol to new year.

Time passed since 2013: Number of months passed from the first of January 2013 to submission of protocol.

SSCCCS: Sample size calculation component composite score.

**Fig. 2:**
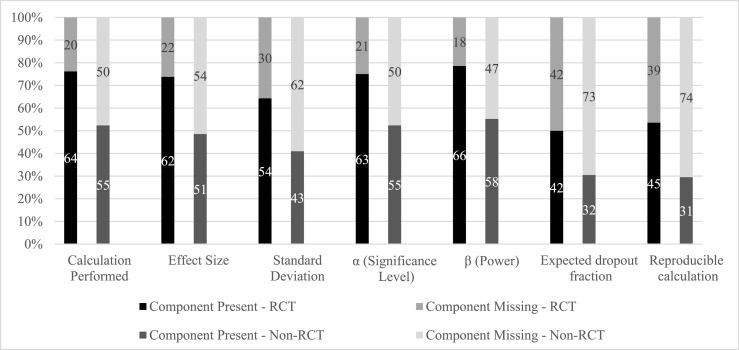
Reported components of sample size calculation. Frequencies of reported components of sample size calculation. The numbers in the columns represent the total number, whereas the Y-axis indicates the percentage. The first, darker column in the pairs represents the RCT, whereas the lighter second in the pairs represents the non-RCTs.

Of the 84 RCTs, 64 (76%) performed a sample size calculation, 62 (74%) reported effect size, 54 (64%) reported standard deviation, 63 (75%) reported α, 66 (79%) reported β, 42 (50%) reported expected dropout fraction, and 45 (54%) contained a sample size calculation reproducible by the authors. Of the 105 non-RCTs 55 (52%) performed a sample size calculation, 51 (49%) reported effect size, 43 (41%) reported standard deviation, 55 (52%) reported α, 58 (55%) reported β, 32 (30%) reported expected dropout fraction, and 31 (30%) contained a calculation reproducible by the authors. The primary endpoint, a comparison between sample size calculations performed in RCT vs. non-RTC study protocols, showed that significantly more RCTs performed a sample size calculation (76% vs 52%, p < 0.001). The mean SSCCCS was 4.0 (SD 2.3) for RCT study protocols and 2.6 (SD 2.4) for non-RCT protocols, p < 0.001 (Fig. 2).

The two most common explanations for not including a sample size calculation in the protocols where sample size calculation was not performed were; missing data (38%), e.g. SD not accessible, and sample size calculation done prior or later (24%), reported in the protocol as some kind of sample size estimation calculation was to be done later or was done prior to the protocol but with no mention of the calculation itself, whereas the rationale for the proposed sample size was often not explained (35%) or based on the investigators research experience (32%) (Table 2).

**Table 2:** Characteristics of study protocols with missing sample size calculation.

	RCT		Descriptive		Experimental		Analytical		Secondary data analysis		Total	
	N = 16		N = 18		N = 13		N = 20		N = 5		N = 72	
Researcher's reason for not including a sample size calculation in the protocol												
Explorative study, N (%)	12	(60.0)	7	(38.9)	6	(46.2)	6	(37.5)	2	(40.0)	33	(45.8)
Incalculable design, N (%)	3	(15.0)	4	(22.2)	2	(15.4)	3	(18.8)	0	(0.0)	12	(16.7)
Calculated prior or later, N (%)	1	(5.0)	3	(16.7)	4	(30.8)	6	(37.5)	3	(60.0)	17	(23.6)
None, N (%)	4	(20.0)	4	(22.2)	1	(7.7)	1	(6.3)	0	(0.0)	10	(13.9)
Researcher's sample size rationale												
Similar studies, N (%)	5	(25.0)	0	(0.0)	1	(7.7)	3	(18.8)	0	(0.0)	9	(12.5)
Experience, N (%)	1	(5.0)	9	(50.0)	5	(38.5)	5	(31.3)	3	(60.0)	23	(31.9)
Predetermined size, N (%)	1	(5.0)	1	(5.6)	2	(15.4)	2	(12.5)	1	(20.0)	7	(9.7)
Calculated prior or later, N (%)	3	(15.0)	3	(16.7)	1	(7.7)	1	(6.3)	0	(0.0)	8	(11.1)
None, N (%)	10	(50.0)	5	(27.8)	4	(30.8)	5	(31.3)	1	(20.0)	25	(34.7)

When sample size was estimated without a calculation, the reason for missing calculation (values: missing data/pilot study/incalculable design/calculated prior or later/none) and the sample size rationale (values: similar studies/experience/predetermined size/calculated prior or later/none) were found in the statistical subsection of the protocol.

Explorative study: some constant or variable (e.g. SD) is unknown, often not specified which in the protocol.

Incalculable design: the sample size calculation deemed ”incalculable” by the investigator due to the study design.

The following extracted variables were statistically significantly associated with having performed a correct sample size calculation in the protocol (Table 3): RCT vs. non-RCTs (OR 2.91, 95% CI 1.55–5.47, p = 0.001), studies with treatment and other intervention vs. studies with no intervention (OR 2.03, 95% CI 1.12–3.69, p = 0.020), studies from the state hospital vs. regional and non-hospital institutions (OR 1.88, 95% CI 1.01–3.49, p = 0.046), studies with foundation donation funding vs. no funding (OR 2.25, 95% CI 1.04–4.87, p = 0.040), studies including blood samples vs. studies without blood samples (OR 1.84, 95% CI 1.01–3.35, p = 0.045), increasing number of sick participants (OR 1.09 per 25 sick participant sample increase, 95% CI 1.00–1.20, p = 0.048) and increasing time passed since 1st of January 2013 (OR 1.04 per month increase, 95% CI 1.00–1.07, P = 0.032).

**Table 3:** Factors associated with having performed a sample size calculation in the included study protocols.

Factor	Calculation performed		OR (95% CI)	P-value
Study type, N (%)	RCT	64 (76.2)	2.91 (1.55–5.47)	0.001
	Non-RCT	55 (52.4)	1.00	
Researcher's academic status, N (%)	Junior	34 (66.7)	1.25 (0.63–2.45)	0.552
	Senior	85 (61.6)	1.00	
Researcher's gender, N (%)	Female	54 (70.1)	1.70 (0.92–3.14)	0.091
	Male	65 (58.0)	1.00	
Institution, N (%)	State hospital	64 (53.8)	1.88 (1.01–3.49)	0.046
	Others	55 (46.2)	1.00	
Intervention, N (%)	Yes	74 (69.2)	2.03 (1.12–3.69)	0.020
	No	43 (52.4)	1.00	
Biobank				
Existing, N (%)	Yes	15 (60.0)	0.87 (0.30–2.57)	0.804
	No	110 (63.2)	1.00	
New, N (%)	Yes	63 (68.5)	1.59 (0.88–2.89)	0.126
	No	56 (57.7)	1.00	
Remuneration, N (%)	Yes	85 (63.9)	0.87 (0.46–1.66)	0.678
	No	109 (64.1)	1.00	
Multistate, N (%)	Yes	10 (52.6)	0.62 (0.24–1.61)	0.325
	No	34 (60.7)	1.00	
Field of medicine, N (%)	Internal medicine	54 (64.3)	1.11 (0.59–2.10)	0.734
	Surgery	15 (62.5)	1.03 (0.40–2,65)	0.945
	Others	50 (61.7)	1.00	
Procedures involved				
Electro-magnetic radiation exposure, N (%)	Yes	23 (62.2)	0.96 (0.46–2.01)	0.958
	No	96 (63.2)	1.00	
Blood sample, N (%)	Yes	74 (69.2)	1.84 (1.01–3.35)	0.045
	No	45 (54.9)	1.00	
Biopsy, N (%)	Yes	12 (60.0)	0.87 (0.34–2.24)	0.772
	No	107 (67.5)	1.00	
None, N (%)	Yes	79 (67.5)	0.60 (0.33–1.10)	0.099
	No	40 (55.6)	1.00	
Economic funding, N (%)	State funded	5 (62.5)	1.77 (0.36–8.65)	0.480
	Medicinal company funded	7 (50.0)	1.06 (0.30–3.71)	0.924
	Foundation donation funded	91 (67.9)	2.25 (1.04–4.87)	0.040
	None	16 (48.5)	1.00	
Study Length (OR per month)			0.01 (0.99–1.02)	0.358
Participants, healthy (OR per 25)			1.01 (0.98–1.05)	0.492
Participants, sick (OR per 25)			1.09 (1.00–1.20)	0.048
Sessions (OR per 1)			0.96 (0.88–1.05)	0.350
Time till/from new year (OR per weeks)			0.99 (0.96–1.04)	0.904
Time passed since 2013 (OR per month)			1.04 (1.00–1.07)	0.032

In linear regression analysis of the SSCCCS RCTs vs. non-RCTs (score increase by 1.38, 95% CI of 0.70–2.06, p < 0.001), studies with female vs. male investigators (score increase by 0.739, 95% CI 0.03–1.45, p = 0.041), studies with treatment and other intervention vs. none (score increase by 0.78, 95% CI 0.08–1.48, p = 0.030), and studies funded by foundations vs. studies with no funding (score increase by 1.39, 95% CI 0.47–2.31, p = 0.003) were associated with a higher score of SSCCCS.

## 4 Discussion

RCT protocols submitted to the Scientific Ethics Committees of The Capitol Region of Denmark were more inclined to include a sample size calculation compared to other study designs. This may be explained by the consensus that research trials including treatments or interventions should be conducted more carefully since the subjects potentially are at a greater risk compared to other study types. An increasing number of projected sick participants in the study also increased the likelihood of including a sample size calculation in the protocol, possibly also explained by the greater respect when the study involves treatment.

Studies funded by a foundation included a sample size calculation more often than studies funded by other means and studies not funded. More strict foundation regulations possibly explain this. Also, investigators pursuing funding with major funding bodies likely pay more attention to important details of their study's design, and consequently studies including realistic and scientifically sound considerations about projected sample size are more likely to obtain funding.

Interestingly, more sample size calculations were performed in newer studies (from 2013 through 2015). This could be explained by more awareness in general among researchers about power and sample size in scientific studies as time passes.

Our study has several strengths: the consecutive inclusion of study protocols during a specified time period led to a random pattern of study characteristics in our sample thus reducing bias. The wide variety of project-specific explanatory variables included increased the information extracted from this study. The centralised structure of the Danish scientific ethics committees ensured a complete coverage of protocols since no local institutional review boards exist. The single, homogenous protocol report formula proved to be an easy way to standardize data, which if multiple formulae types were involved could induce confusion and misunderstanding. Conversely, it is a limitation of the study that it only included a small sample of protocols in a limited geographical area.

Ideally, planning of all studies with quantitative analysis involving human subjects should include a sample size estimation [7]. However, some study designs make sample size calculations difficult, specifically pilot studies within a field so unexplored that no constants can be used in the calculation [8]. The interpretation of studies with inadequate or missing sample size calculations could potentially differ greatly from studies with correct calculation of sample size. Studies with too few research subjects risk failing to reject the null hypothesis, but also risk accepting a false hypothesis that otherwise would have been rejected if additional research subjects had been enrolled. In contrast, studies with an abundancy of research subjects unethically waste funds and subject additional participants to unnecessary hazard without a clear justification.

Our investigation confirmed that estimation of sample size is more often performed in RCTs than non-RCT studies. We extend previous findings by showing that several project-specific factors increase the probability of reporting a sample size calculation in scientific protocols. Particularly, intervention studies, studies funded by a foundation donation, studies conducted at the state hospital, studies with blood samples drawn, studies with a high number of sick participants and chronologically newer studies are more likely to report a sample size calculation. This study increases the knowledge within a sparsely studied area and highlight the importance of adequate sample size estimation in academic research projects. The knowledge generated by this study is important for researchers from academia as well as members of scientific ethics committees and institutional review boards when evaluating the impact and soundness of research. Further, it ultimately may prevent the initiation of unjustified and unsound research that exposes patients to unnecessary harm and possibly delays important knowledge from being adopted by the research community and in clinical practice.

## Conflicts of interest

None.

## Funding

None.
